# ARETT: Augmented Reality Eye Tracking Toolkit for Head Mounted Displays

**DOI:** 10.3390/s21062234

**Published:** 2021-03-23

**Authors:** Sebastian Kapp, Michael Barz, Sergey Mukhametov, Daniel Sonntag, Jochen Kuhn

**Affiliations:** 1 Department of Physics, Technische Universität Kaiserslautern, Erwin-Schrödinger-Str. 46, 67663 Kaiserslautern, Germany; mukhamet@physik.uni-kl.de (S.M.); kuhn@physik.uni-kl.de (J.K.); 2 German Research Center for Artificial Intelligence (DFKI), Interactive Machine Learning Department, Stuhlsatzenhausweg 3, Saarland Informatics Campus D3_2, 66123 Saarbrücken, Germany; michael.barz@dfki.de (M.B.); daniel.sonntag@dfki.de (D.S.); 3 Applied Artificial Intelligence, Oldenburg University, Marie-Curie Str. 1, 26129 Oldenburg, Germany

**Keywords:** augmented reality, eye tracking, toolkit, accuracy, precision

## Abstract

Currently an increasing number of head mounted displays (HMD) for virtual and augmented reality (VR/AR) are equipped with integrated eye trackers. Use cases of these integrated eye trackers include rendering optimization and gaze-based user interaction. In addition, visual attention in VR and AR is interesting for applied research based on eye tracking in cognitive or educational sciences for example. While some research toolkits for VR already exist, only a few target AR scenarios. In this work, we present an open-source eye tracking toolkit for reliable gaze data acquisition in AR based on Unity 3D and the Microsoft HoloLens 2, as well as an R package for seamless data analysis. Furthermore, we evaluate the spatial accuracy and precision of the integrated eye tracker for fixation targets with different distances and angles to the user (n=21). On average, we found that gaze estimates are reported with an angular accuracy of 0.83 degrees and a precision of 0.27 degrees while the user is resting, which is on par with state-of-the-art mobile eye trackers.

## 1. Introduction

Head mounted displays (HMD) got more affordable and lightweight in the last few years facilitating a broader usage of virtual and augmented reality (VR/AR) applications. In addition, recent devices are equipped with integrated eye trackers which primarily target novel gaze-based interaction techniques [1,2] and optimizing the display quality, e.g., using foveated rendering [3,4]. This creates new opportunities for eye tracking research in mixed reality settings. However, the number and functionality of research tools for AR and VR eye tracking is still limited, e.g., compared to the well-established stationary eye trackers that are attached to a two-dimensional display. Available commercial solutions for HMD eye tracking are mostly limited to VR (see, e.g., References [5,6]). Pupil Labs [6] offers an extension for AR eye tracking which consists of mobile eye tracking equipment attached to an HMD, but with only a loose integration into AR application development tools.

In this work, we aim at closing the gap of research tools for AR eye tracking. We implement an open-source toolkit that facilitates eye tracking research in AR environments with the Microsoft HoloLens 2. Our toolkit includes a package for the Unity 3D game development engine which enables simple integration of reliable gaze and meta data recordings in AR applications, and an R package for seamless post-hoc processing and analysis of the data. In addition, we conduct a user study (n=21) for evaluating the spatial accuracy and precision of the gaze signal retrieved from our toolkit. We discuss our results and compare them to results for state-of-the-art mobile eye trackers from the literature.

## 2. Related Work

Our work is related to other research-oriented toolkits and software solutions for head-mounted eye tracking systems, particularly to those targeting VR and AR environments, and to literature on measuring the gaze estimation error.

### 2.1. AR and VR Eye Tracking

Some toolkits for eye tracking research in VR are available. Tobii offers a solution for eye tracking analysis in VR by providing tools for the integration of eye tracking hardware to HMDs and analysis software for eye tracking research [7]. Another commercial eye tracking add-on is offered by Pupil Labs for the HTC Vive HMD together with open-source software for data analysis [6]. Non-commercial frameworks for eye tracking in AR or VR exist, as well. Stratmann et al. [8] presented EyeMR, a low-cost system for integrating eye tracking into VR based on the Pupil Capture software and a custom Unity 3D framework. Lee et al. [9] also presented a method for low-cost gaze tracking and gaze point estimation in head-mounted devices. Mardanbegi and Pfeiffer [10] presented the EyeMRTK toolkit to develop gaze-based interaction techniques in VR and AR; however, the current implementation is limited to specific VR headsets. Adhanom et al. [11] presented the GazeMetrics tool which provides a standardized approach to measure accuracy and precision in VR settings.

The range of AR eye tracking toolkits is more limited. Pupil Labs [6] offers eye tracking add-ons for the Microsoft HoloLens 1 and the Epson Moverio BT-300, but the analysis software is tailored to mobile eye tracking without HMDs and their integration into the Unity 3D development environment is discontinued (https://github.com/pupil-labs/hmd-eyes/issues/100#issuecomment-662362737, accessed on 20 November 2020). This limits the usefulness of the offered add-ons and restricts applications to use cases in which no AR integration is required. Recent HMDs, like the Magic Leap 1 [12] and the Microsoft HoloLens 2 [13], are equipped with integrated eye trackers. However, the toolkits and APIs provided by the manufacturers are targeted at gaze-based interaction and not at eye tracking research [14,15]. Still, this enables an easy integration of visual attention into AR applications: using the spatial awareness of the devices provides eye-in-world data which otherwise has to be integrated using additional sensors [16]. We build our toolkit on top of the eye tracking APIs of the HoloLens 2 device [13]. However, all device specific code is encapsulated in a data access layer which enables easy adaption of the toolkit to other eye tracking enabled AR devices.

### 2.2. Measuring the Gaze Estimation Error

Eye tracking research studies investigate the impact of an intervention on the eye movements of a participant. Typically, the gaze samples or fixations, i.e., the periods for which the eye is relatively still, are used to approximate the human visual attention, and are mapped to areas of interest (AOIs) for analysis. High gaze estimation quality is essential for eye tracking research because errors can heavily undermine the results [17]. However, a key problem in head-mounted eye tracking is that the gaze estimation error, i.e., the difference between the estimated and true gaze position, can be substantial, particularly if participants move and if fixation distances vary [18,19]. Besides user position and orientation, also factors specific to the eye tracker and display, e.g., parameters of the calibration routine and of the display detection algorithm, can have significant impact on the gaze estimation error [20]. Typical metrics for the error of gaze estimation include spatial accuracy and spatial precision [21]. Spatial accuracy is commonly computed as the mean angular deviation of fixations to the actual position, and spatial precision as the root mean square error or standard deviation of individual gaze samples from their centroid [21,22].

## 3. Augmented Reality Eye Tracking Toolkit

We develop an eye tracking toolkit for augmented reality applications using the Unity 3D game development engine [23]. Our goal is to simplify the access to eye tracking data from the Microsoft HoloLens 2 for research purposes or advanced interaction techniques. We aim at providing raw gaze data robustly at a fixed data rate, without delay, and with highest possible spatial accuracy and precision. For this, we implement an easy-to-use interface to control recordings and enable a simple integration into applications and research studies. In addition, we implement a package for the statistical computing environment R for seamless data analysis [24]. The toolkit, a detailed documentation, and an example project are available on GitHub (https://github.com/AR-Eye-Tracking-Toolkit/ARETT, accessed on 22 March 2021) under the MIT open-source license.

### 3.1. Overview of HoloLens 2 Technology

We briefly summarize the underlying technology, i.e., the eye tracking hardware and software of the HoloLens 2 which we interface in our data access layer. Similar to other head-mounted eye trackers, the Microsoft HoloLens 2 uses two infrared cameras that yield a close-up view of the wearer’s eyes [13]. After using the built-in 9-point calibration routine, a closed processing module provides real-time 3D gaze data to developers including a gaze origin and a direction vector. Gaze data can be accessed within Unity 3D via the Mixed Reality Toolkit (MRTK) [14] and via the underlying API for the Universal Windows Platform (UWP) [25]. The MRTK primarily focuses on enabling gaze-based interaction via an easy-to-use API for developers. It does not offer recordings for research purposes, nor does it guarantee a fixed sampling rate which is tied to the Unity3D update rate. Hence, gaze samples might be missed. Our system is based on the API for the UWP which provides unsmoothed data, more stable data rates, and a higher level of control. Further, a high precision timestamp in the system-relative QueryPerformanceCounter (QPC) time domain with a precision of 100 ns is provided for each data point. The manufacturer is vague in reporting specifications related to data quality: the data rate is “approximately 30 Hz” with a spatial accuracy that ranges “approximately within 1.5 degrees” [26].

### 3.2. Architecture & Components of the Recording Tool

**Figure 1:**
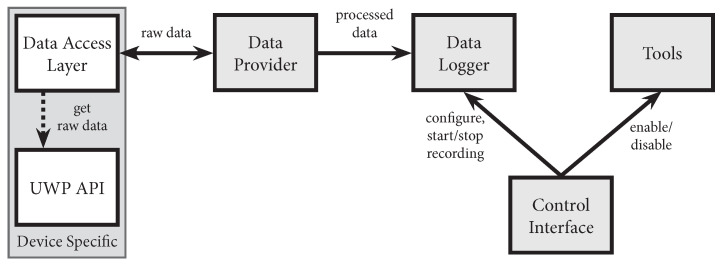
A diagram visualizing the components of the toolkit and their interaction.

The recording tool of our toolkit is implemented as a package for the Unity 3D game development engine and includes four major components: the generic data provider with the HoloLens-specific data access layer that makes timestamped gaze data available in real-time, the data logger that is responsible for storing the data, the web-based control interface, and a set of utility tools for data visualization. An overview of our system’s architecture and the interplay of individual components is shown in Figure 1. In the following, we describe each component in detail and discuss the implementation of egocentric video capture.

The data provider accesses raw eye tracking data using the data access layer, processes it and raises according gaze data events. The data access layer on the HoloLens 2 checks for new gaze samples in a separate thread every 10 ms to reliably obtain all gaze samples from the API with a supposed data rate of 30 Hz, i.e., we expect a new gaze sample every 33.33 ms. This pulling is necessary as no new data event is provided by the API. Each gaze sample includes the origin of the gaze point, its direction vector, and a timestamp. All gaze samples received by the access layer are queued in the data provider and processed in the next frame update in the Unity 3D main thread. For each gaze sample, we cast a ray and check for hits with collider objects in the scene. If the option spatial mapping of the MRTK is enabled for the application, this includes the real environment that is scanned by the depth sensors of the HoloLens 2. If a collider is hit, we extend the gaze sample by the intersection coordinates in the world coordinate system, the object’s name, position, rotation and scale, the intersection point in the object’s local coordinate system, and the gaze point projection to the 2D eye displays. In addition, we support AOI colliders for real-time gaze-to-AOI mapping with support for dynamic AOIs. AOI collider objects can be placed at any position of a Unity 3D scene or attached to virtual objects in the scene. AOIs must be defined during the application development phase. Real-time and gaze-based adaptations can be realized using custom scripts. Synchronized recordings of the gaze signal and the front-facing camera can be used to define further AOIs post-hoc. We separately cast gaze rays to check for hits with AOI colliders. In addition, we offer an option to store the position, rotation and scaling of game objects in the scene in our gaze sample. This can be used to simulate or visualize sequences of interest post-hoc. For each processed sample, we raise an event that can be subscribed by other components, such as the data logger.

The data logger component provides the option to record all gaze samples. An overview of all recorded data columns can be found in Table 1. The files are named based on the participant’s pseudonym and a custom recording name. All recordings of a participant are stored in one folder. The gaze data samples are saved as comma separated values (CSV) with one sample per row and the columns as described in Table 1. In addition, we store meta information of the recording, e.g., the start and end time of the recording, in a separate text file in the JSON format. After the recording is started, developers can log additional events in terms of an info string that is stored as part of the gaze sample and in the JSON file. This enables researchers to track custom interaction events, which are of interest to their research question, and session annotations. The recording can be started via function calls and is used in our web-based control interface.

We integrate two utility tools that ease the development, debugging, and monitoring of study prototypes. This includes a tool for visualizing a grid of fixation targets, and one for highlighting AOIs. The grid of fixation targets enables easy collection of gaze samples and corresponding target positions for the evaluation of spatial accuracy and precision. We use this tool in our evaluations: we show nine fixation targets arranged in a 3×3 grid at multiple distances from the user. The AOI highlighting helps in debugging dynamic and interactive experiment scenes in which AOIs can move around, appear and disappear during the experiment session. For this, the developer can add custom visualizations which can be dynamically shown and hidden using the control interface.

**Figure 2:**
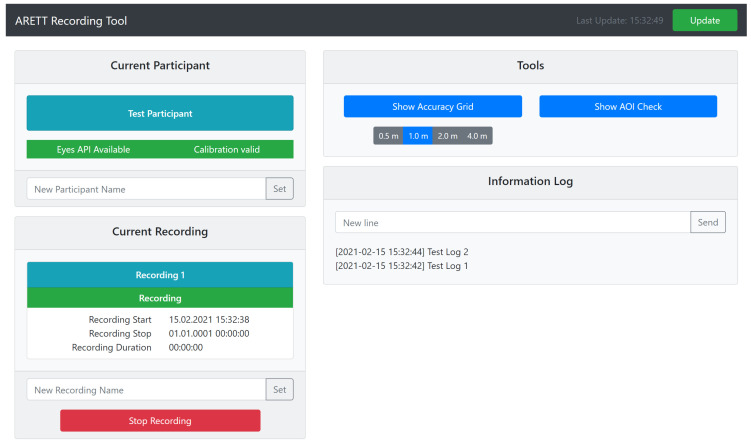
Screenshot of the control interface accessible over the network.

Our toolkit comes with a web-based control interface (see Figure 2). It enables the experimenter to easily set a participant acronym and a recording name, and to start and stop recordings from any computer in the local network. Further, it provides access to our utility tools and allows an experimenter to add custom annotations to the recording during the study.

Typically, head-mounted eye trackers use a world camera to record the environment from an egocentric perspective and map the wearer’s pupil positions to the corresponding video frames. The integrated eye tracker of the Microsoft HoloLens 2, however, maps pupil positions to gaze rays in the 3D coordinate system of the device.

Our toolkit adds a virtual camera to the 3D scene that matches the location, projection, and resolution of the integrated front-facing camera. This enables the projection of the 3D gaze position to the virtual 2D camera image and, hence, to the webcam image. The virtual camera is preconfigured to match the integrated, front-facing webcam of the HoloLens 2. We recommend to check the configuration per use case and to adapt it, if the camera specifications differ. The 2D gaze signal is reported via the gaze sample event of the data provider and recorded in the *gazePointWebcam* column.

If video streaming or capturing for demonstration purposes is required only, the Mixed Reality Capture (MRC) module of HoloLens 2 can be used. It streams or records an egocentric video with an overlay showing the virtual content [27]. Our toolkit supports gaze visualization in this module by attaching a small sphere to the current gaze position that is visible in the capture but not to the user. However, this method is computationally demanding which constrains the framerate for all applications to 30 frames per second and has a negative impact on real-time interactive applications which limits its use to demonstration purposes.

### 3.3. R Package for Data Analysis

We implement an R package for seamless data analysis of recordings from our recording tool. Existing data analysis tools are primarily targeted at stationary eye trackers that yield a two-dimensional gaze signal or mobile eye trackers that report gaze with respect to an egocentric video feed [5,28,29,30]. Our toolkit reports three dimensional gaze data with a world-centered coordinate system. We provide a new R package that supports this data paradigm. It offers offline fixation detection with corresponding pre- and post-processing routines. The R package and detailed documentation is published on GitHub (https://github.com/AR-Eye-Tracking-Toolkit/ARETT-R-Package, accessed on 22 March 2021) under the MIT open-source license.

We implement two functions for pre-processing the raw gaze data, *gap fill* and *noise reduction*, similar to Reference [31]. The *gap fill* function linearly interpolates between valid gaze points with small gaps in between, e.g., due to loss of tracking. The *noise reduction* function applies a mean or median filter to the gaze data with a given window size.

Three methods from the literature for offline fixation detection are implemented. This includes I-VT using a velocity threshold similar to Reference [31], I-DT for VR as described by Llanes-Jurado et al. [32] using a dispersion threshold, and I-AOI proposed by Salvucci and Goldberg [33] based on detected areas of interest. Our implementation of I-VT follows the description by Olsen [31]. It reproduces a similar behavior based on the data recorded using our toolkit. We calculate a velocity for each gaze point over a specified duration and categorize the points by comparing the velocities to a specified threshold. I-DT follows the implementation by Llanes-Jurado et al. [32]. It computes the angular dispersion distance over a window of a specific size in terms of its duration. If the initial window exceeds this threshold it is moved forward until it does not exceeded the threshold. Then, the window is extended to the right until the dispersion threshold is exceeded. All samples in the window, excluding the last sample, are classified as belonging to a fixation. Afterwards, a new window is initialized at the position of the last gaze sample. These steps are repeated until all samples are classified. The I-AOI method for fixation detection is based on Salvucci and Goldberg [33]. It differs from the other methods as it classifies fixations based on predefined areas of interest. First, all gaze points within an AOI are classified as belonging to a fixation. Next, groups of fixation samples are identified as a fixation event using a minimum duration threshold. Short events are discarded.

In addition, we provide two functions for post-processing of detected fixations: *merging adjacent fixations* and *discarding short fixations*. The *merge adjacent fixations* function merges subsequent fixations if the gap is smaller than a defined maximum duration and, depending on the detection algorithm used, a maximum angle between them (I-VT) or a maximum dispersion distance (I-DT). For I-AOI, the two fixations must belong to the same AOI. The *discard short fixations* function removes short fixations based on a minimum fixation duration and is mainly interesting for the I-VT method because both other methods inherently contain a minimum fixation duration.

## 4. Evaluation of Accuracy and Precision

High eye tracking data quality is important for eye tracking research because errors in the gaze estimation process can undermine the validity of reported results [17]. However, for the integrated eye tracker of the Microsoft HoloLens 2 we only find limited information about spatial accuracy and no information about spatial precision [26]. We conduct a user study to analyze the accuracy and precision of gaze data from the HoloLens 2 that is recorded using our toolkit. We ask participants to fixate a set of targets, which have a static position with respect to the participant’s head, at different distances. We record the gaze signal while the participants are seated (setting I) or walking (setting II). Further, we ask them to fixate a target with a static world position while moving around (setting III). The results can serve as a reference for researchers when designing eye tracking studies, e.g., to decide whether the accuracy is sufficient, or to influence the position and size of AOIs. In addition, our results can guide interaction designers that develop gaze-based AR applications, for example to improve gaze-based selection [34].

### 4.1. Participants

In total, we recruited 21 participants (7 or 33% female; mean age 29.5, SD=8.5) of which 15 participated in all three settings. Two participants skipped setting III and four participants finished setting III only. This totals to 17 participants for settings I and II (4 or 24% female; mean age 29.1, SD=8.6), and 19 participants for setting III (7 or 37% female; mean age 30, SD=8.8). All participants had normal or corrected-to-normal vision with one participant wearing contact lenses and three participants wearing glasses.

### 4.2. Conditions & Tasks

**Figure 3:**
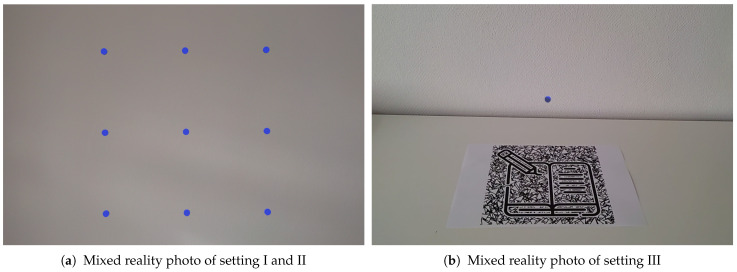
Mixed reality photo of our HoloLens 2 applications for all three settings which are presented to the participants. The fixation grid for settings I and II is displayed at a fixed distance from the user and resized such that the angular size is identical for all distances (**a**). The sphere in setting III is positioned 15 cm above the table and stays fixed on top of the visual marker when the participant moves (**b**). These screenshots are 2D projections which do not reflect the field-of-view and depth perception of a participant in augmented reality (AR).

**Figure 4:**
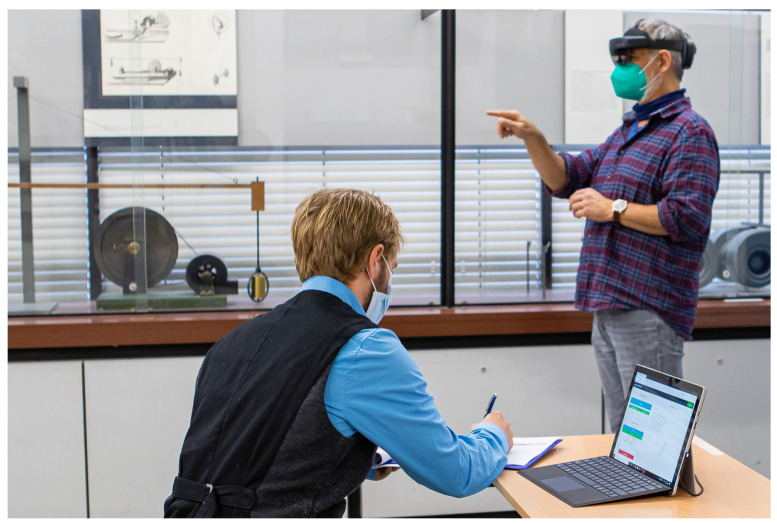
Example of setting I and II in our study with the participant wearing a Microsoft HoloLens 2 and the supervisor controlling the recording using our toolkit.

In our study, we include three settings in which we record the participants’ gaze signal and the position of multiple fixation targets. In setting I and II, we show a planar 9-point grid of fixation targets (3×3) that is centered in front of the participant’s head and orthogonal to the forward direction (Figure 3a). Participants are standing still in setting I, and walking forward and backward in setting II during the recording phase. For both settings, the grid size is aligned to the field of view of the device. The outer fixation targets are positioned at the border of the field of view such that both eyes can see them. The distances between the corner targets (upper left, upper right, lower left, lower right) and the center target are 18.25 degrees of visual angle. The distances for the edge targets (upper center, middle left, middle right, lower center) are 12.13 degrees of visual angle. In addition, we vary the distance *d* of the grid for both settings: we include d∈{0.5m,1m,2m,4m}. For all distances, we ask the participants to fixate all targets for three seconds, starting on the upper left in a left-to-right and top-to-bottom direction. An example picture of the settings I and II can be found in Figure 4. For setting III, we place a single fixation target at a static position in the world coordinate system: we show a sphere with diameter of 1 cm 15 cm above the surface on a table with a height of 75 cm (Figure 3b). Participants are seated in front of the table and are asked to move their heads left and right while keeping up the fixation to the sphere. With this setting, we simulate vestibulo-ocular reflex movements that are common in natural experiment settings in which participants interact with stationary AR content.

### 4.3. Procedure

All settings are recorded in one session, starting with setting I and immediately followed by setting II and III. The order of the settings was identical for all participants. In the beginning of a session, the participant puts on the device which is adjusted to the head by a supervisor. The device is fitted to a participant’s head such that it does not move during the experiment but is still comfortable to wear. If the participant feels that the device loosens, it is tightened by the supervisor. During the whole procedure, the device is not moved on or removed from the participant’s head. After fitting, the integrated eye tracker is calibrated using the built-in calibration routine. We record gaze data and reference target positions with our new toolkit. Each task is recorded separately, resulting in a recording per distance for setting I and II, and a single recording for setting III. For settings I and II, we perform a manual fixation detection and remove gaze samples that belong to a saccade event. We performed a manual annotation of the gaze signal to extract fixations more accurately than possible with automatic algorithms which have, in particular, problems with event detection in mobile eye tracking signals [35]. Gaze samples are labeled as belonging to a fixation unless the gaze position moved away from the fixation center, i.e., when turning into a saccade which ends at the next fixation center. The labeling is based on visual inspections from one expert. For setting III, we remove gaze samples before the participant starts fixating the sphere and moving his/her head, and after the participant stops. The participant is asked by the supervisor to start the movement and, after four minutes, asked to stop moving and to return to the starting position.

### 4.4. Metrics

We define spatial accuracy and precision according to the literature [34,36]. Per target, we compute spatial accuracy as the distance between the mean gaze sample and the target position. Spatial precision is computed as the standard deviation of the distances between each gaze sample and the mean position of all gaze samples. We report both measures in cm, as well as in degrees of visual angle. The distance in cm is calculated using the distance between the gaze point and the target based on their positions in the reference frame provided by Unity 3D. The visual angle is calculated as the angle between the reported 3D gaze ray from the gaze origin to the gaze point and the 3D ray from the gaze origin to the target position.

### 4.5. Hypotheses

Previous research on the gaze estimation error in head-mounted eye tracking reported significant differences in the spatial accuracy for varying distances and when moving around versus resting [18,20]. We expect similar characteristics for the integrated eye tracker of the Microsoft HoloLens 2. Hence, we hypothesize that the spatial accuracy is dependent on the distance of the fixation target (H1). Further, we expect a lower accuracy for setting II in which participants move than for setting I in which they are resting (H2). Similar to H2, we expect that spatial precision is lower for setting II, i.e., when participants move (H3). For setting III, we exploratively investigate the spatial accuracy and precision for a realistic research setting from educational sciences.

### 4.6. Results

A total of 335,867 gaze points are recorded over all participants in all three settings and before filtering. Analyzing the relative timestamp provided by the device, the mean difference between timestamps is 33 ms (SD 1 ms). One hundred and seventy-one of these gaze points show a time difference to the previous gaze point larger than 34 ms, and 27 gaze points show a difference smaller than 32 ms. Those with a difference larger than 34 ms are multiples of the expected 33.33 ms. All gaze points with a difference smaller than 32 ms have a difference of 0 ms. After removing the 198 gaze points with erroneous timing, we see a mean difference between timestamps of 33.33 ms (SD $2.5\times 10^{-4}$ ms).

**Figure 5:**
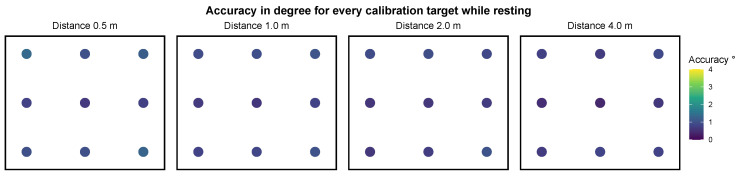
Plot of the mean accuracy at each distance for each target in setting I—resting. The accuracy angle for all targets is smaller than 1.5 degrees.

**Figure 6:**
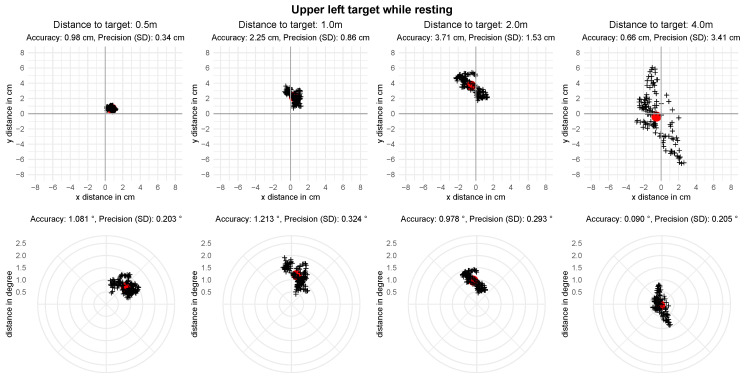
Recorded gaze point of one participant in relation to the upper left target in setting I—resting. The red dot represents the mean gaze position with each cross being one recorded gaze point.

For setting I, we report the metrics for all targets which include, on average, 108.47 (SD=43.04) gaze points after saccade removal. Table 2 shows the spatial accuracy and precision per distance, averaged over all nine fixation targets and participants. The mean angular accuracy over all distances is 0.83 degrees with a precision of 0.27 degrees. Figure 5 visualizes the error for individual targets per distance. A visualization of the analyzed gaze positions of one participant at the upper left target can be found in Figure 6. A Shapiro-Wilk test shows that the means of accuracies in degrees of visual angle over all targets is not distributed normally for all distances but 2.0 m, $p_{0.5}=0.01$, $p_{1.0}=0.03$, $p_{2.0}=0.12$, $p_{4.0}=0.03$. To evaluate the difference in spatial accuracy over all distances we conduct a Friedman test. It shows a significant difference in accuracy between the different distances, $\chi^{2}\left(3\right)=20.15$, p<0.001. Post hoc analysis with Wilcoxon signed-rank tests is conducted with a Bonferroni correction applied, resulting in a significance level set at p<0.008. It reveals a significant difference in the accuracy between the distance 0.5 m and the distances 2.0 m and 3.0 m (Table 3).

**Figure 7:**
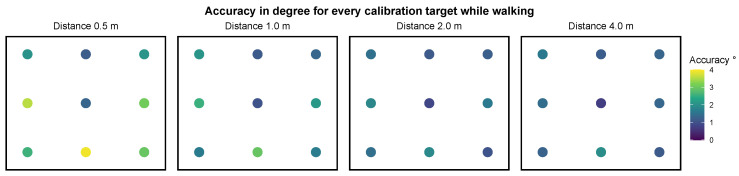
Plot of the mean accuracy at each distance for each target in setting II—walking.

**Figure 8:**
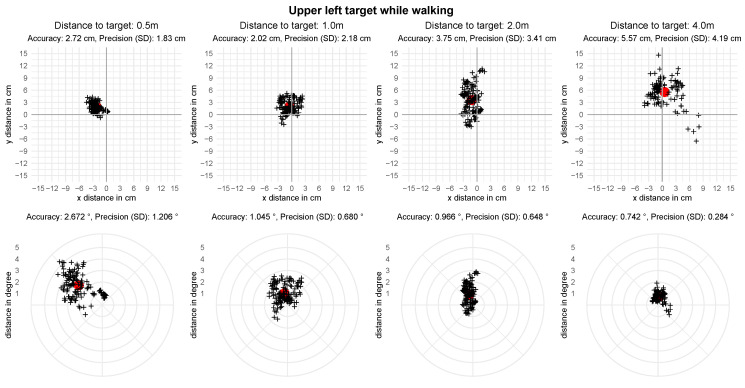
Recorded gaze point of one participant in relation to the upper left target in setting II—walking. The red dot represents the mean gaze position with each cross being one recorded gaze point.

The recordings for setting II include an average of 121.23 (SD=32.53) gaze samples per target. The mean spatial accuracy, averaged over participants and fixation targets per distance, is reported in Table 4. The mean angular accuracy over all distances is 1.77 degrees with a precision of 1.13 degrees. The results per fixation target are visualized in Figure 7. A visualization of the analyzed gaze positions of one participant at the upper left target can be found in Figure 8. The mean accuracy in degrees of visual angle over all targets is distributed normally for the distances 0.5 m and 4.0 m, but not at 1.0 m and 2.0 m as assessed by a Shapiro-Wilk test, $p_{0.5}=0.44$, $p_{1.0}=0.01$, $p_{2.0}=0.04$, $p_{4.0}=0.35$. Analogue to setting I we conduct a Friedman test to evaluate the difference in spatial accuracy over all distances. It shows a significant difference in accuracy between the different distances, $\chi^{2}\left(3\right)=37.02$, p<0.001. The Bonferroni corrected post hoc analysis with Wilcoxon signed-rank tests results in a significance level set at p<0.008. It reveals a significant difference in spatial accuracy for all paired comparisons except for the distances 2.0 m and 4.0 m (Table 5).

In addition, we compare the spatial accuracy and precision results between setting I (resting) and setting II (walking). The differences in accuracy are not distributed normally for the distances 0.5 m and 1.0 m as assessed by a Shapiro-Wilk test, $p_{0.5}=0.04$, $p_{1.0}=0.003$, $p_{2.0}=0.26$, $p_{4.0}=0.44$. A Wilcoxon signed-rank test shows that the accuracy differs significantly between setting I and II for all distances (Table 6). The difference in precision is distributed normally for the distance of 0.5 m but not for the other distances as assessed by a Shapiro-Wilk test, $p_{0.5}=0.44$, $p_{1.0}<0.001$, $p_{2.0}=0.046$, $p_{4.0}=0.003$. A Wilcoxon signed-rank test shows that the precision differs significantly between setting I and II for all distances (Table 7).

**Figure 9:**
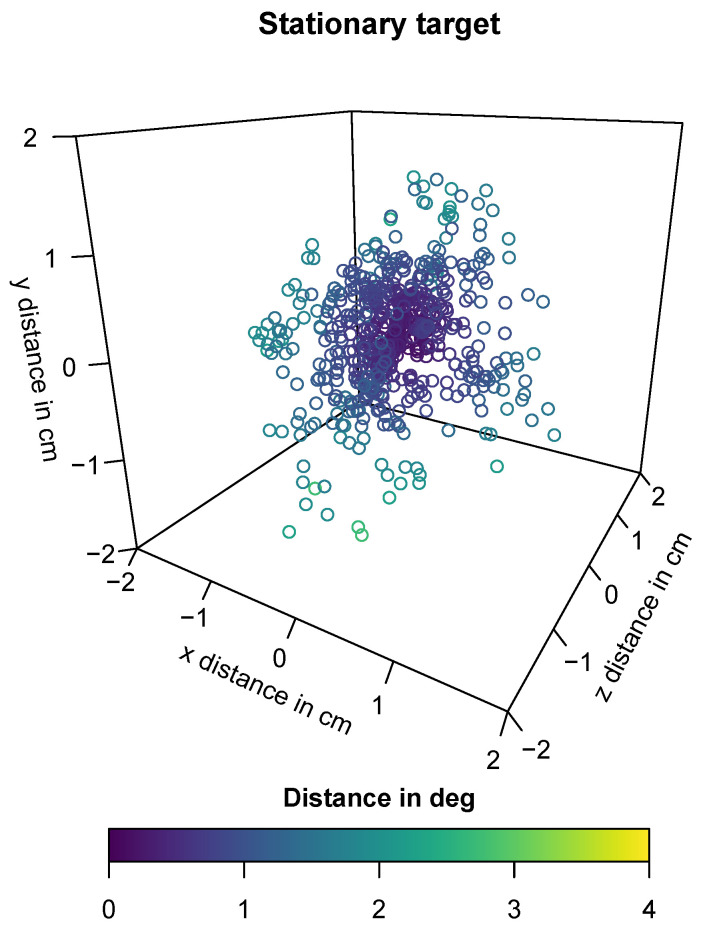
Recorded gaze point of one participant in setting III—stationary target. The distance angle for all gaze points is smaller than 3 degrees.

For setting III, we include a mean of 641.79 (SD=262.10) gaze samples per participant for our analysis. The resulting accuracy and precision values together with the mean distance of the participants from the target can be found in Table 8. We approximate the spatial accuracy in degrees of visual angle as using the following formula: $\theta =tan^{-1}\left(O/d\right)$ with the accuracy in cm as *O* and the mean distance to the participant *d*. The same formula is used to calculate the precision by using the precision in cm as *O*. A 3D visualization of the analyzed gaze positions of one participant can be found in Figure 9.

## 5. Discussion

The major goal of developing the augmented reality eye tracking toolkit is to enable researchers to easily use eye tracking in AR settings with the HoloLens 2. It should allow an efficient integration to Unity 3D scenes, enable recordings of a comprehensive set of eye tracking signals (see Table 1), and a seamless analysis of the data via our R package. This would simplify integration of eye tracking into existing AR research, like Strzys et al. [37] and Kapp et al. [38]. Independently from the study reported in this publication, our toolkit is currently being used in two ongoing research studies which provide first evidences in this direction. One study utilizes the Microsoft HoloLens 2 to display two dimensional plots at a fixed distance while the participant is moving while another study investigates stationary augmentations on a table. The initial feedback from the study organizers, the developers of the AR application, and the experimenters is positive. No major issues occurred during the recordings, which certifies a high robustness, and the ease-of-use of the web interface was, informally, rated high.

Our toolkit can also be used for facilitating gaze-based interaction and real-time adaptive applications using the data provider module. For instance, prior research proposed to use eye tracking and HMDs to augment the episodic memory of dementia patients by storing artificial memory sequences and presenting them when needed [39]. Other works include approaches for gaze-based analysis of the users’ attention engagement and cognitive states for proactive content visualization [40], and multi-focal plane interaction, such as object selection and manipulation at multiple fixation distances [41]. It can also be used in research regarding selection techniques in AR [42,43]. The utility of the toolkit for realizing real-time adaptive applications has been shown in Reference [44]. The presented prototype uses video and gaze information via our toolkit to automatically recognize and augment attended objects in an uninstrumented environment.

### 5.1. Evaluation of Accuracy and Precision

The results from our evaluation show significant differences in spatial accuracy for varying distances in setting I and II. This supports our hypothesis H1. However, for setting I, the pairwise comparisons reveal that only the results for the smallest distance 0.5 m and the distances 2.0 and 4.0 m differ significantly. For setting II, the results significantly differ for all pairs except for the two farthest distances of 2.0 m and 4.0 m. Further, our results confirm the hypothesis H2 and H3: the accuracies and precision for each distance differ significantly between setting I and setting II while the results for setting II are poorer.

Our observations also show that the spatial accuracy in degrees of visual angle increases with increasing distance (see Table 2 and Table 4). Findings from the literature suggest that the accuracy decreases with increasing deviation from the calibration distance, i.e., the distance at which the fixation targets of the calibration routine are shown [18,20,45]. This leads to our assumption that the built-in calibration routine of HoloLens 2 is placed at 2 to 4 m from the user, which is supported by the fact that Microsoft recommends an interaction distance of 2 m [46]. It is possible that this increase in angular accuracy is an effect of the vergence-accommodation conflict [47] as only a combined gaze ray is made available by the device.

The official HoloLens 2 documentation reports a vague range for the spatial accuracy of “approximately within 1.5 degrees” with “slight imperfections” to be expected [26]. Basically, our results coincide with these specifications, but are much more fine-grained. For the resting setting (I), we observe better spatial accuracy values ranging from 1.00 degrees of visual angle for a 0.5 m distance to 0.68 degrees for 4.0 m. For the walking setting (II), which has a lower spatial accuracy overall, the results for 0.5 m and 1.0 m are outside the official range with 2.52 and 1.84 degrees of visual angle, respectively. The two other conditions lie within the specified boundary of 1.5 degrees. The documented sampling rate of “approximately 30 Hz” was also met with a new gaze sample being observed every 33.33 ms.

Based on our findings, we suggest minimum target sizes for eye tracking research and gaze-based interaction with the HoloLens 2. Similar to Feit et al. [34], who investigate the gaze estimation error for remote eye tracking, we calculate the minimum size such that 95% of all gaze samples hit the target. We use their formula that computes the minimum size based on a 2-dimensional Gaussian function as S=2(O+2σ) with the spatial accuracy of the eye tracker as offset *O* and the spatial precision of the gaze signal as σ. The resulting minimum target sizes for varying distances are listed in Table 9. For a distance of 2.0 m, Microsoft recommends a target size of 5–10 cm, which conforms with our findings for setting I: we suggest a target size of 11.10 cm in this case. However, if the participant is meant to move around, the targets should be significantly larger.

In setting III, we explore the characteristics of the gaze estimation error for stationary targets. The average distance to the stationary target of 49.87 cm is comparable to the 0.5 m distance in setting I. However, the mean spatial accuracy is better and the precision is lower. This better result for spatial accuracy could be explained by the longer fixation durations and the varying viewing angles in setting III: on average, the mean gaze positions seem to balance around the fixation target, while the dispersion stays high (see Figure 9). Based on Feit et al. [34], we suggest a minimum target size of 4.16 cm. This is 22% larger than the recommendation for setting I, and 34% of the recommended size for setting II. Altogether, the results suggest that the fixation duration and the user condition, i.e., walking versus not walking, influences the spatial accuracy and precision, which should be considered when designing interactive and, potentially, mobile research applications.

Finally, we compare the results of the HoloLens 2 eye tracker to available head-mounted eye trackers without an HMD. Macinnes et al. [48] evaluated the spatial accuracy and precision of three mobile eye trackers for multiple distances while participants were seated. They included (i) the Pupil Labs 120 Hz Binocular glasses with an accuracy of $0.84^{\circ }$ and a precision of $0.16^{\circ }$, (ii) the SensoMotoric Instruments (SMI) Eye Tracking Glasses 2 with an accuracy of $1.21^{\circ }$ and a precision of $0.19^{\circ }$, and (iii) the Tobii Pro Glasses 2 with an accuracy of $1.42^{\circ }$ and a precision of $0.34^{\circ }$. On average, our results for the HoloLens 2 in setting I, which is the closest match to the setting in Reference [48], yield an accuracy of $0.83^{\circ }$ and a precision of $0.27^{\circ }$. This is similar to the results of the Pupil Labs glasses that ranged best in the experiment by Macinnes et al. [48] and suggests that the eye tracking data from HoloLens 2 can effectively be used in research experiments. However, one drawback is that the sampling rate of 30 Hz is lower compared to the devices evaluated in their experiment.

### 5.2. Limitations

Our toolkit enables access to raw gaze data and provides additional tools for processing them. However, it is limited to the data that is made available through APIs of the device. For instance, the API reports a joint gaze vector for both eyes, while many commercial binocular eye tracking glasses report separate gaze rays. This forces to intersect the gaze ray with the virtual environment to receive a point of gaze. Separate rays can be intersected to extract gaze points without intersecting any surface, and to infer the fixation depth. In addition, this gaze point can be used to find close-by AOIs. Our evaluation focuses on limited set of interaction settings that probably do not generalize to all possible settings in AR environments. However, with setting III, we include a more realistic setting that closer matches typical AR environments with a moving user and fixed visualizations. We cannot rule out effects due to the experiment order as it was identical for all participants.

Currently, our toolkit is constrained to the Microsoft HoloLens 2 as eye tracking device. However, all device specific functionality is encapsulated in the data access layer. This makes it possible to adapt the toolkit to other eye tracking enabled AR devices in the future. However, the gaze estimation is device-specific: the results from our evaluation on spatial accuracy and spatial precision do not hold for other devices. In addition, the sampling rate might change which needs to be addressed by re-configuring the data pulling rate. The data access layer could also subscribe gaze events or connect to a signal stream, if this is supported by the new device.

## 6. Conclusions

In this work, we presented an open-source toolkit that enables eye tracking research in AR using the HoloLens 2 device. We addressed the gap of missing research tools by implementing a Unity 3D package for reliable gaze data acquisition and an R package for seamless data analysis. We received first positive feedback on our toolkit from two other research studies, proving its utility. We conducted a user study (n=21) to investigate the spatial accuracy and spatial precision of gaze data from our toolkit. The results suggest that the spatial accuracy increases when increasing the distance of fixation targets. Further, we found evidence that spatial accuracy and precision drop when participants are walking compared to standing still. Overall, the gaze estimation error is similar to recent head-mounted eye trackers without HMDs which shows the suitability of our toolkit for research applications. In future updates we will address the limitations of our toolkit as follows. We plan to add fully integrated support for video recording of the integrated camera using the data logger, as well as real-time streaming of video and gaze data. We will also investigate the effectiveness of attaching virtual AOIs to real objects for real-time gaze-to-AOI mapping. Further, we want to extend the functionality of our R package and integrate interfaces to existing gaze data processing tools, as well as integrate data access layers for other devices.

## Figures and Tables

**Table 1:** Overview of recorded data.

Data Column	Description
Time data	
eyeDataTimestamp	Unix timestamp of the gaze data (in ms)
eyeDataRelativeTimestamp	Relative timestamp of the gaze data (in ms, 100 ns precision)
frameTimestamp	Unix timestamp of the frame in which the data was processed (in ms)
Gaze data	
isCalibrationValid	Flag if the calibration of the wearer is valid
gazeHasValue	Flag if valid gaze data exists (origin/direction)
gazeOrigin_(x/y/z)	Gaze origin in the global reference frame
gazeDirection_(x/y/z)	Gaze direction in the global reference frame
gazePointHit	Flag if the raycast hit an object and a gaze position exists
gazePoint_(x/y/z)	Position of the gaze point in the global reference frame
gazePoint_target_name	Name of the game object hit by the gaze ray
gazePoint_target_(x/y/z)	Position of the gaze point in the local reference frame of the hit object
gazePoint_target_(pos/rot/scale)_(x/y/z)	Position, rotation, and scale of the game object hit by the gaze ray
gazePoint(Left/Right/Mono)Screen_(x,y,z)	Position of the gaze point on the left, right and virtual mono display
gazePointWebcam_(x,y,z)	Position of the gaze point on the webcam image
AOI data	
gazePointAOIHit	Flag if the gaze ray hit an AOI
gazePointAOI_(x/y/z)	Position of the gaze point on the AOI in global coordinates
gazePointAOI_target_name	Name of the game object representing the AOI
gazePointAOI_target_(x/y/z)	Position of the gaze point in the local reference frame of the AOI
gazePointAOI_target_(pos/rot/scale)_(x/y/z)	Position, rotation, and scale of the game object hit by the AOI ray
gazePointAOIWebcam_(x,y,z)	Position of the gaze point on the AOI on the webcam image
Additional information	
gameObject_*objectName*_(pos/rot/scale)_(x/y/z)	Position, rotation, and scale of selected game objects
info	Info string of a logged event

**Table 2:** Accuracy and precision for setting I—resting.

Distance	Accuracy (SD)		Precision (SD)	
	in cm	in deg	in cm	in deg
0.5 m	0.91 (0.41)	1.00 (0.44)	0.40 (0.16)	0.29 (0.13)
1.0 m	1.56 (0.83)	0.85 (0.46)	0.67 (0.24)	0.25 (0.11)
2.0 m	2.85 (1.31)	0.77 (0.35)	1.35 (0.49)	0.24 (0.10)
4.0 m	5.03 (2.27)	0.68 (0.31)	3.12 (1.26)	0.28 (0.12)

**Table 3:** Results of the post hoc Wilcoxon signed-rank tests for setting I—resting. * the Bonferroni corrected significane level is p<0.008.

Comparison	0.5 m	0.5 m	0.5 m	1.0 m	1.0 m	2.0 m
	−1.0 m	−2.0 m	−4.0 m	−2.0 m	−4.0 m	−4.0 m
Z	−2.63	−3.57	−3.43	−1.68	−2.06	−1.44
p	0.009	<0.001 *	0.001 *	0.093	0.039	0.149

**Table 4:** Accuracy and precision for setting II—walking.

Distance	Accuracy (SD)		Precision (SD)	
	in cm	in deg	in cm	in deg
0.5 m	2.29 (0.64)	2.52 (0.69)	1.89 (0.34)	1.31 (0.25)
1.0 m	3.35 (1.50)	1.84 (0.81)	3.33 (1.00)	1.16 (0.47)
2.0 m	5.07 (1.94)	1.39 (0.53)	6.32 (1.52)	1.03 (0.27)
4.0 m	9.75 (3.08)	1.33 (0.42)	12.58 (3.19)	1.03 (0.32)

**Table 5:** Results of the post hoc Wilcoxon signed-rank tests for setting II—walking. * the Bonferroni corrected significance level is p<0.008.

Comparison	0.5 m	0.5 m	0.5 m	1.0 m	1.0 m	2.0 m
	−1.0 m	−2.0 m	−4.0 m	−2.0 m	−4.0 m	−4.0 m
Z	−3.432	−3.621	−3.621	−3.574	−2.817	−0.686
p	0.001 *	<0.001 *	<0.001 *	<0.001 *	0.005 *	0.492

**Table 6:** Results of the Wilcoxon signed-rank tests for the comparison of the accuracy between setting I and II.

Distance	0.5 m	1.0 m	2.0 m	4.0 m
Z	−3.62	−3.62	−3.57	−3.53
p	<0.001	<0.001	<0.001	<0.001

**Table 7:** Results of the Wilcoxon signed-rank tests for the comparison of the precision between setting I and II.

Distance	0.5 m	1.0 m	2.0 m	4.0 m
Z	−3.62	−3.62	−3.62	−3.62
p	<0.001	<0.001	<0.001	<0.001

**Table 8:** Accuracy, precision, and mean distance for setting III—stationary target.

Distance (SD)	Accuracy (SD)		Precision (SD)	
in cm	in cm	in deg	in cm	in deg
49.87 (13.53)	0.34 (0.27)	0.39 (0.31)	0.87 (0.35)	1.00 (0.40)

**Table 9:** Recommended minimum target size in cm based on Feit et al. [34] and the identified accuracy and precision.

Distance	Setting I (Resting)	Setting II (Walking)
0.5 m	3.42 cm	12.14 cm
1.0 m	5.80 cm	20.02 cm
2.0 m	11.10 cm	35.42 cm
4.0 m	22.54 cm	69.82 cm

## Data Availability

The data presented in this study are available on request from the corresponding author. The data are not publicly available due to data privacy.
